# Mouse models for gastric cancer: Matching models to biological questions

**DOI:** 10.1111/jgh.13297

**Published:** 2016-07-27

**Authors:** Ashleigh R Poh, Robert J J O'Donoghue, Matthias Ernst, Tracy L Putoczki

**Affiliations:** ^1^ Department of Medical BiologyUniversity of MelbourneMelbourneVictoriaAustralia; ^2^ The Walter and Eliza Hall Institute of Medical ResearchMelbourneVictoriaAustralia; ^3^ School of Cancer MedicineLa Trobe University, Olivia Newton‐John Cancer Research InstituteMelbourneVictoriaAustralia

**Keywords:** diffuse gastric cancer, *Helicobacter pylori*, intestinal‐type gastric cancer, mouse model

## Abstract

Gastric cancer is the third leading cause of cancer‐related mortality worldwide. This is in part due to the asymptomatic nature of the disease, which often results in late‐stage diagnosis, at which point there are limited treatment options. Even when treated successfully, gastric cancer patients have a high risk of tumor recurrence and acquired drug resistance. It is vital to gain a better understanding of the molecular mechanisms underlying gastric cancer pathogenesis to facilitate the design of new‐targeted therapies that may improve patient survival. A number of chemically and genetically engineered mouse models of gastric cancer have provided significant insight into the contribution of genetic and environmental factors to disease onset and progression. This review outlines the strengths and limitations of current mouse models of gastric cancer and their relevance to the pre‐clinical development of new therapeutics.

## Introduction

Gastric cancer (GC) is the third leading cause of cancer mortality worldwide and affects approximately 500 000 individuals per year.^1^ With the exception of Japan, most GC patients are diagnosed at advanced stages of the disease because of the lack of screening available for early detection.^2^ Conventional treatments include surgery, chemotherapy, and radiation therapy; however, recurrence occurs in 50% of patients with a 5‐year survival rate of approximately 20%.^3^ Thus, early detection and monitoring of tumor responses to treatment are central to improving patient survival, while a deeper understanding of tumor‐specific molecular targets is necessary to facilitate the development of novel and more effective therapeutics.

Chemical‐induced and genetically engineered mouse models to induce random and targeted genetic mutations, respectively, have revolutionized our understanding of how genes, diet, infection, and carcinogens affect GC. Likewise, these models have provided an invaluable platform for drug discovery and validation. However, to fully exploit these models, an in‐depth understanding of their tumor histopathology, molecular signatures, and the molecular networks driving the disease is important to ensure that there is an optimal match between the questions investigated and the models used for this purpose. Here, we endeavor to provide an updated description of the most commonly used GC models together with a discussion of their strengths and limitations.

## Classification of GC subtypes

Gastric adenocarcinomas may be subdivided into two main histological groups according to the Lauren classification.^4^ However, due to the high level of molecular heterogeneity in GC, there has been an emerging interest in further classifying GC based on patterns of molecular alterations and their correlation with disease progression and prognosis.^5^, ^6^

### Lauren classification of GC

**Figure 1:**
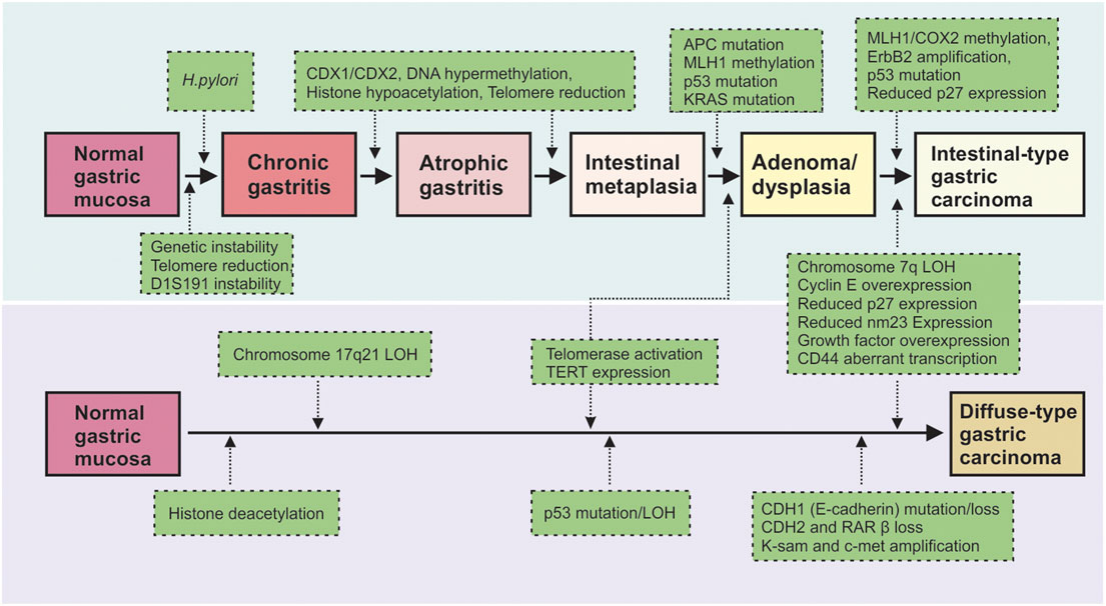
Pathogenesis of intestinal‐type and diffuse‐type gastric cancer. Intestinal‐type gastric cancer (top half of diagram) develops through a series of well‐characterized events, whereby *Helicobacter pylori* infection promotes the differentiation of the normal gastric mucosa to chronic gastritis, which then progresses to atrophic gastritis. This may eventually result in the formation of dysplastic lesions and intestinal‐type gastric carcinoma. In contrast, the molecular mechanisms underpinning diffuse‐type gastric carcinoma (bottom half of diagram) are less well defined, but are commonly associated with mutations or loss of E‐cadherin. *Figure adapted from Yuasa (2003).*

The Lauren classification of GC was first proposed in 1965 and comprises the well‐differentiated intestinal and the poorly differentiated diffused types. These subtypes differ not only in their morphology but also in terms of epidemiology, pathology, and genetic profiles. Intestinal‐type GC is often initiated by *Helicobacter pylori* infection, which induces chronic gastritis and atrophy (Fig. 1).^7^ Atrophy is characterized by the loss of specialized glandular tissue including the oxyntic glands with parietal cells, which modulate growth and differentiation of gastric progenitor cells.^8^ In addition to the loss of important differentiation signals, a reduction in parietal cells also leads to a decrease in gastric acid secretion and achlorhydria. Consequently, bacterial overgrowth can occur, which promotes inflammation and other pathophysiological changes.^7^ Indeed, gastrin‐deficient mice, which have achlorhydric stomachs, are characterized by bacterial overgrowth and chronic atrophic gastritis that progresses to GC.^9^ Likewise, *Helicobacter felis*‐infected wild‐type mice also develop achlorhydria, atrophy, and gastric tumors.^10^

As a consequence of chronic inflammation and glandular regeneration, metaplasia often occurs in intestinal‐type GC. At least two distinct subtypes of metaplasia have been identified, namely, intestinal‐type and spasmolytic polypeptide‐expressing metaplasia (SPEM).^11^ Intestinal‐type metaplasia is characterized by an increase in goblet‐like cells and the expression of mucin (MUC)‐2 and trefoil factor (TFF)3, while SPEM is identified by the presence of TFF2 and MUC‐6 expressing cells that histologically resemble cells of the Brunner's glands.^11^ Interestingly, while the relationship between *H. pylori* infection, atrophy, and intestinal metaplasia is well established, a causal relationship between intestinal metaplasia and neoplastic transformation is somewhat more tenuous.^12^ Indeed, SPEM is more strongly associated with GC progression and is therefore thought to be a precursor to the development of cancerous lesions.^13^, ^14^ Intestinal‐type adenocarcinomas exhibit well‐defined glandular structures with features such as multiple lumens, reduced stroma, and enlarged nuclei, and these adenocarcinomas are typically surrounded by a dense population of inflammatory immune cell infiltrates.^4^, ^15^ The metaplasia–neoplasia–carcinoma sequence is driven by additional mutations in genes, including *TP53,* 16 *APC,* 17 *RB1*,^18^ and *TGFβ*,^19^ which collectively control cellular survival and proliferation. The role of these genes in the neoplastic transformation of the gastric mucosa has been expertly reviewed by others.^15^, ^20^

Compared with intestinal‐type GC, the carcinogenic pathway driving the development of diffuse‐type GC is less well characterized. Diffuse adenocarcinomas appear as solitary clusters with a poorly differentiated and disorganized epithelial structure. The epithelial cells within these clusters are often rich in mucous‐containing vacuoles that displace the nucleus to the periphery to form “signet‐ring” cells, a characteristic of malignancy.^2^ Although diffuse adenocarcinomas can be initiated by *H. pylori* infection, they are more commonly associated with defects in the cell‐adhesion protein E‐cadherin (Fig. 1), because mutation, deletion, and methylation of the corresponding *CDH1* gene is frequently observed in human disease.^4^, ^21^ E‐cadherin plays a key role in epithelial integrity, and loss of E‐cadherin expression is associated with disruption of cell–cell junctions and impairment of cell adhesiveness.^22^ Furthermore, impaired E‐cadherin activity has also been associated with increased cell growth and invasion of tissues adjacent to epithelial cancer cells, indicating a tumor suppressive role of *CDH1.* 23, 24 Because of defects in adhesion, diffuse‐type adenocarcinomas exhibit a higher frequency of invasion and metastasis compared with the intestinal subtype and are therefore considered to be more aggressive.^25^

### Molecular classification of distinct GC subtypes

**Figure 2:**
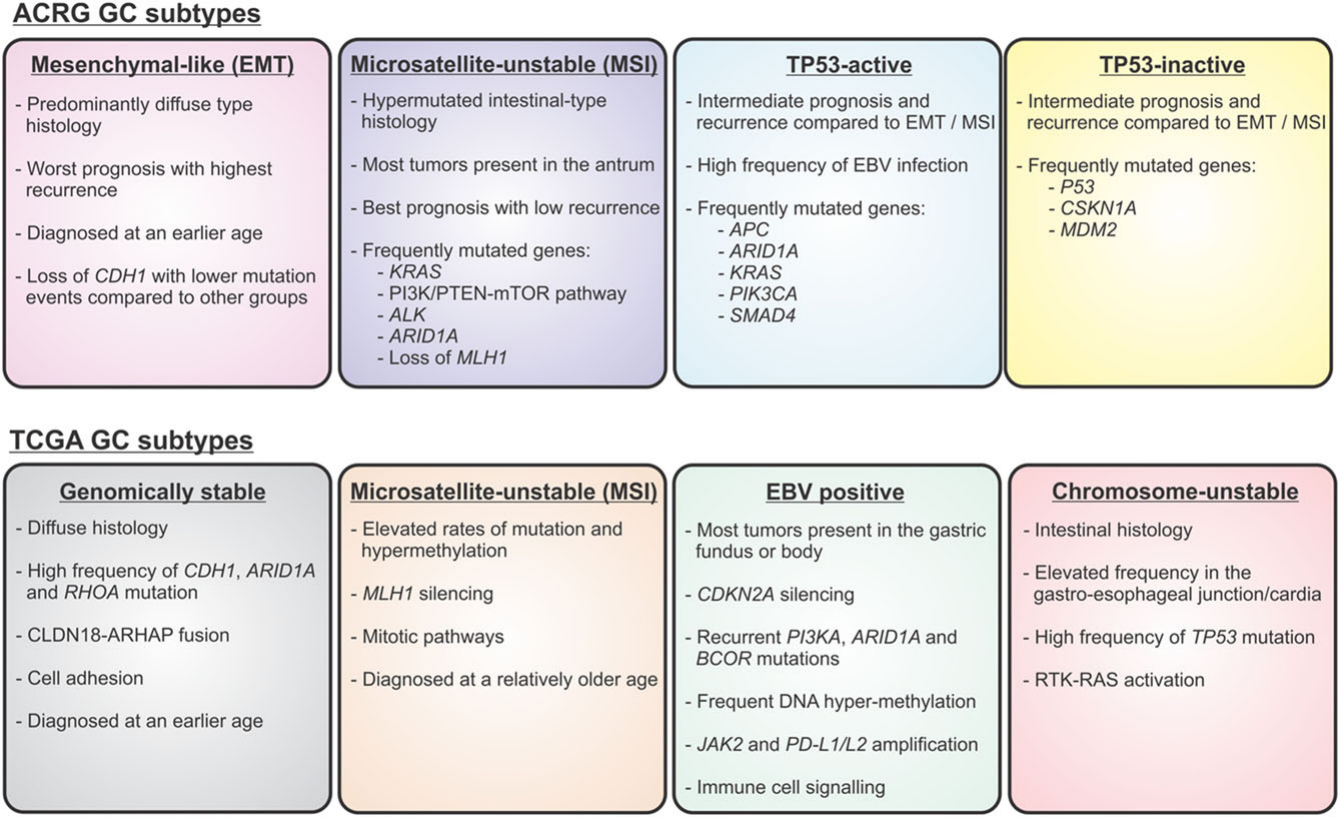
Molecular characterization of gastric cancer subtypes. Comprehensive molecular characterization of gastric tumors has identified four main subtypes associated with distinct patterns of genetic alterations, disease progression, and clinical prognosis. The four subtypes identified by the Asian Cancer Research Group (top half of diagram) include the mesenchymal‐like type (EMT), microsatellite‐unstable (MSI), and tumor protein 53 (TP53)‐active and TP53‐inactive subtypes. The four subtypes defined by the The Cancer Genome Atlas (bottom half of diagram) include tumors positive for Epstein–Barr virus, microsatellite unstable tumors, genomically stable tumors, and tumors with chromosomal instability.

While the Lauren classification has been useful for evaluating GC epidemiology and etiology, this morphological approach for classifying gastric tumors offers only limited clinical utility and does not predict response to available treatments. Thus, recent studies have used gene expression profiling and the availability of somatic genome‐wide information to identify several distinct molecular subtypes over and above the Lauren classification (Fig. 2).^5^, ^6^ For instance, the Asian Cancer Research Group (ACRG) profiled 251 primary gastric tumors in terms of gene expression and gene copy number and combined this information with targeted gene sequencing to define four distinct GC molecular subtypes that were associated with distinct tumor recurrence and clinical prognosis. These subtypes include the mesenchymal‐like type (EMT), microsatellite‐unstable (MSI) groups, alongside the tumor protein 53 (TP53)‐active and TP53‐inactive subtypes (Fig. 2).^6^ The EMT subtype is observed in a significantly younger subset of patients than any of the other subtypes, where the majority of patients (>80%) are diagnosed with diffuse‐type tumors at stage III/IV and have a high frequency of recurrence (~63%). Moreover, while tumors in the EMT group are often characterized by loss of *CDH1*, overall, they have a lower number of mutation events when compared with the other groups.^6^ In contrast, MSI tumors exhibit intestinal‐type features and predominantly occur in the antral region of the stomach (75%). These tumors are often hyper‐mutated and carry mutations in genes of the PI3K‐PTEN‐mTOR pathway (42%), as well as in *ARID1A* (44.2%), *KRAS* (23.3%), or *ALK* (16.3%). MSI tumors are typically diagnosed at an early stage (I/II) and have the best prognosis with lowest recurrence.^6^ Lastly, the TP53‐active and TP53‐inactive groups exhibit an intermediate prognosis and recurrence compared with EMT and MSI subtypes. Interestingly, TP53‐active tumors are also associated with a highest probability of Epstein–Barr virus infection. On the other hand, the TP53‐inactive subtype has a high prevalence of TP53 mutation (60%) but exhibit low mutations in *CSKN1A* and *MDM2*, while TP53‐active tumors exhibit a higher prevalence of mutations in *APC*, *ARID1A*, *KRAS*, *PIK3CA*, and *SMAD4.* 6

A complementing assessment of 295 primary gastric adenocarcinomas by The Cancer Genome Atlas (TCGA) project also identified four distinct subtypes (Fig. 2). Again, an Epstein–Barr virus positive group was identified and exhibited recurrent *PIK3CA* mutation, DNA hyper‐methylation, and amplification of *JAK2* and *PD‐L1/L2*. The MSI tumors in the TGCA dataset also showed elevated mutation rates in genes involved in oncogenic signaling pathways. However, this project also identified genomically stable tumors, which are enriched for the diffuse histologic variant, and tumors with chromosomal instability that exhibit aneuploidy and amplification of receptor tyrosine kinases.^5^ Interestingly, comparison of ACRG and TCGA datasets revealed a number of similarities, including the enrichment of TCGA genomically stable tumors in ACRG EMT subtypes and TCGA EBV positive groups in ACRG TP53‐active subtypes, respectively.^5^, ^6^ However, a number of differences were also observed, such as the presence of TCGA tumors classified as chromosomal or genomically unstable in all ACRG subtypes. Furthermore, no equivalent subtypes that corresponded to the ACRG TP53‐active and TP53‐inactive groups were identified in the TCGA cohorts.^5^, ^6^ Thus, while molecular characterization studies are crucial for laying the groundwork towards the development of personalized medicine, an overall consensus remains to be established to more definitely classify clinically relevant subtypes.

While these genetic subtypes most likely help to stratify patients to new targeted therapeutics, they have not been fully replicated by mouse models. Accordingly, most of the mouse models described in the succeeding discussion have been evaluated most extensively based on the histological appearance of their cancer lesions, with only some having been assessed on the basis of similar gene expression pattern between histologically similar tumors in mice and humans.

## MNU‐induced gastric carcinogenesis

Prior to the classification of *H. pylori* infection as a type I carcinogen, chemical agents were used to induce GC in mice. The best characterized model of chemically induced GC involves administration of N‐methyl‐N‐nitrosourea (MNU), an N‐nitroso compound generated by anaerobic gut bacteria following ingestion of nitrates and nitrites. Normally MNU is supplemented in drinking water, and its tumorigenic efficacy depends on concentration rather than total intake.^26^ Treatment of mice with MNU at 120 ppm during 5 alternating weeks promotes cancer development in a large number of genetic backgrounds (Table 1),^27^ and this concentration is therefore recommended in most standard protocols. When used in conjunction with a high‐salt diet, a known risk factor for GC, MNU‐dependent mutagenesis significantly increases the frequency of tumor development.^28^ MNU‐induced tumors occur predominantly in the gastric antrum and mostly comprise well‐differentiated to moderately differentiated adenocarcinomas with a large stromal cell component.^27^

**Table 1:** GC susceptibility to MNU mutagenesis in different mouse strains

Strain	Incidence (affected/total) (%)	Adenocarcinoma (affected/incidence)			Depth of invasion (affected/incidence)				Ref.
		WD	PD	SC	M	S	MP	SS	
BALB/c	20/27 (74)	18	1	1	2	12	3	—	Yamachika *et al.*,^26^ Yamamoto *et al.*,^27^ and Tatematsu *et al.* 165
C3H/HeN	7/26 (26)	3	4	—	5	2	—	—	Yamamoto *et al.* 27 and Tatematsu *et al*.^123^
C57/Bl6	8/26 (30)	8	—	—	2	6	—	—	Yamamoto *et al.* 27 and Nakamura *et al*.^166^
CBA/JN	7/23 (30)	6	1	—	5	1	—	—	Yamamoto *et al.* 27
CD‐1 (ICR)	6/27 (22)	4	2	—	2	3	—	1	Yamamoto *et al.* 27
DBA/2N	6/29 (20)	5	1	—	2	4	—	—	Yamamoto *et al.* 27

GC, gastric cancer; M, mucosa; MNU, N‐methyl‐N‐nitrosourea; MP, muscularis propria; PD, poorly differentiated; Ref., references; SC, signet cell type; S, submucosa; SS, subserosa; WD, well differentiated.

The MNU model has revealed a number of signaling pathways and transcription factors involved in gastric tumorigenesis, including p53,^29^ NF‐κB,^30^ mitogen‐activated protein kinase (MAPK),^31^ COX2,^28^, ^32^ *β*‐catenin,^32^ RUNX3,^33^ E‐cadherin,^34^ and KLF‐4.^35^ Although the precise mechanism underpinning MNU‐induced carcinogenesis remains unclear, chromatin remodeling as a result of amino acid modification in histone proteins,^36^ and altered expression of the gastric tumor suppressor *Tff1* has been observed.^37^ Because histone methylation and epigenetic silencing of the promoter for *TFF1* is also frequently detected in human GC, it has been proposed that MNU may mediate tumor development through a series of epigenetic modifications to the TFF1 promoter.

## *Helicobacte*r infection models

*Helicobacter pylori* are gram‐negative, spiral‐shaped bacteria that thrive in the acidic environment of the gastric mucosa.^38^ Infection with *H. pylori* is regarded as the most prominent risk factor for GC and is estimated to be responsible for at least 75% of human gastric adenocarcinomas.^39^ However, while *H. pylori* colonizes the gastric epithelium of at least half of the world's population, only 1–3% of those infected will develop GC.^40^, ^41^ Factors attributing to this include duration of infection, bacterial pathogenicity,^42^ polymorphisms in the host genome,^43^, ^44^ and diet, which collectively influence the extent and magnitude of the immune response. In particular, polymorphisms in inflammatory genes have attracted a considerable amount of attention, because infected patients carrying the *Il1β‐511T* or *Il1β‐31C* alleles not only exhibit augmented levels of IL‐1*β* 45, 46 but also have a higher risk of developing GC.^47^, ^48^ However, other epidemiological studies have revealed less clear links between *Il1β* polymorphisms and GC risk^49^, ^50^ and suggested a much larger number of susceptibility loci as well as environmental factors. Accordingly, models of long‐term infections with *H. pylori* used in conjunction with the introduction of additional preconceived mutations and/or chemical agents have been useful for gaining insight into the molecular mechanisms underpinning *H. pylori*‐mediated gastric carcinogenesis, at least in experimental mice (Table 2).

**Table 2:** Tumor characteristics in *Helicobacter*‐infected mice

Model	AD onset (months)	AD incidence (%)	Location	Phenotype				Ref.
				AT	MT	DP	AD	
*H. felis*	18	80	Corpus	+	+	+	+	Fox *et al*.^54^
*H. felis* + MNU	9	100	Antrum	+	+	+	+	Tomita *et al*.^37^
*H. felis* + INS‐GAS	8	85–100	Corpus	+	+	+	+	Fox *et al*.^108^
*H. felis* + IL1*β*	12	<70	Corpus + antrum	+	+	+	+	Tu *et al*.^119^
*H. pylori* + INS‐GAS	8	100	Corpus	+	+	+	+	Fox *et al*.^75^ and Fox *et al*.^108^
*H. pylori* + MNU	20	<80	Antrum	+	+	+	+	Nakamura *et al*.^166^ and Han *et al*.^167^
*H. pylori* + MNU + high salt	10	100	Antrum	+	+	+	+	Toyoda *et al*.^76^

+, present; AD, adenocarcinoma; AT, atrophy; DP, dysplasia; MNU, N‐methyl‐N‐nitrosourea; MT, metaplasia; Ref., references.

### Helicobacter felis

Due to the resistance to colonization by *H. pylori* in mice,^51^, ^52^ *Helicobacter* infection models frequently exploit *H. felis*, a bacterial strain closely related to *H. pylori*, but derived from the gastric mucosa of cats.^53^ Acute colonization of the mouse stomach with *H. felis* induces severe gastritis, and long‐term infection often results in the development of atrophic lesions that form invasive adenocarcinomas.^10^ Extensive dysplastic lesions are evident in the gastric corpus at the squamocolumnar junction after 12 months of infection,^54^, ^55^ while polyploid antral tumors are typically observed after 2 years and are pathologically similar to those observed in human patients.^53^, ^55^, ^56^, ^57^ Importantly, many of the early effects are reversible, because antibiotic treatment has been shown to reduce inflammation, restore normal gut architecture, and prevent the development of adenocarcinomas.^10^, ^58^ In line with these findings, *H. pylori* eradication in patients with gastritis is the most effective way to decrease their risk of developing GC.^59^

### *H. pylori* SS1 Sydney strain

A number of mouse‐adapted *H. pylori* strains have now been developed, including the well‐characterized Sydney strain (SS1).^60^ After about 8 months of infection with this strain, mice develop severe gastritis and atrophy of the gastric epithelium, which may progress to high‐grade dysplasia, although adenocarcinomas do not usually develop.^60^, ^61^ For this reason, *H. pylori* SS1 is typically used in conjunction with chemical agents or in genetically engineered mice to enable and promote gastric carcinogenesis. The inability of *H. pylori* SS1 to induce adenocarcinomas on its own is thought to be attributed to the loss of its *cag* pathogenicity island (CagA), which encodes a type IV secretion system that enables the bacterium to inject CagA, peptidoglycan, and other proteins into the host cell.^62^ These latter proteins trigger the activation of signaling pathways comprising NF‐*κ*B, MAPK and STAT3, thereby promoting the production of inflammatory cytokines and chemokines.^63^, ^64^ Consistent with this, infection with CagA‐positive *H. pylori* SS1 (PMSS1) induces rapid progression along the intestinal‐type GC progression cascade.^65^

A reduction in *TFF1* gene expression is observed in 50% of human distal GCs and is associated with promoter hyper‐methylation and the corresponding silencing of the *TFF1* gene.^66^, ^67^ Other factors that regulate TFF1 expression include gastrin, which stabilizes TFF1 and suppresses MNU‐induced antral gastric tumor development.^27^, ^37^ Interestingly, *TFF1* is also epigenetically repressed in *H. pylori*‐infected cells,^68^ and *H. pylori* lipopolysaccharide associate with the TFF1 protein dimer *in vitro.* 37, 69, 70 Recently, loss of TFF1 was shown to promote *H. pylori*‐induced *β*‐catenin activation and the development of invasive adenocarcinomas in *Tff1* ^−/−^ mice, suggesting that the loss of TFF1 could be a critical step in promoting *H. pylori*‐mediated oncogenic activation of *β*‐catenin and gastric tumorigenesis.^71^

### Advantages and limitations

*Helicobacter* infection models provide a robust and reproducible means to study immune responses during infection and chronic gastritis. For example, the increased susceptibility of C57BL/6 relative to BALB/c mice to *H. felis*‐induced and *H. pylori* SS1‐induced atrophies^60^, ^72^ has been attributed to a bias towards a T‐helper‐1 (Th1)‐like immune response in C57BL/6 mice that is characterized by an increase in IFN‐*γ* and other pro‐inflammatory cytokines.^72^ Meanwhile, BALB/c mice display a bias towards Th2‐like immune response characterized by increased IL‐4, IL‐10, and other cytokines. The critical role of cytokines in promoting *Helicobacter*‐dependent gastritis severity and duration has also been demonstrated following infection of immune‐deficient mice or of strains that overexpress IL‐10^73^ or IL‐4.^74^

Mouse models of chronic *Helicobacter* infection have also highlighted the contribution to gastric carcinogenesis made by gender, diet, and the commensal microbiota. For instance, *H. pylori* infection of INS‐GAS mice, a strain transgenically expressing gastrin from a rat insulin promoter fragment (see succeeding discussion), confers a greater gastric tumor incidence in males than in females, consistent with human epidemiological studies indicating a higher prevalence of GC in men.^75^ Likewise, the carcinogenic link between infection and dietary salt and nitrates/nitrites intake has been demonstrated in C57/BL6 mice, which develop more pronounced gastric atrophy and a greater risk of adenocarcinomas following infection with *H. pylori* and a high‐salt diet.^76^ Finally, because gastric atrophy results from infection associated with the outgrowth of commensal bacteria that is enabled by the loss of acid‐producing cells, the composition of the gut microbiota is considered another risk factor for GC. In line with this, infection studies using mice housed under germ‐free conditions revealed that the lack of the commensal gut flora in *H. pylori*‐infected INS‐GAS mice reduced gastritis and delayed intraepithelial neoplasia.^77^ Given that complete protection from intraepithelial neoplasia was not observed in this model, this indicates that the gastric microbiota has a tumor‐promoting function rather than tumor‐initiating function. In contrast, limitations of *Helicobacter* mouse models include the scarcity of *Helicobacter* strains and the extensive latency period of *H. pylori*‐induced gastric atrophy and GC. Furthermore, there is a low incidence rate of advanced GC, and while *H. pylori* infection is an established causative factor of duodenal ulcers in humans, mouse models of *H. pylori* infection‐induced duodenal ulcers have not been reported.^78^

### Translational opportunities using *Helicobacter* infection models

The eradication of *H. pylori* in patients with established chronic infections is challenging. Current treatment strategies are focused on long‐term maintenance therapy to prevent infection recurrence and typically include combining acid inhibition with a proton pump inhibitor and at least two antibiotics. However, increasing antibiotic resistance and associated re‐infection with *H. pylori* remains a significant hurdle in the clinic. *Helicobacter* infection models have been a useful tool for identifying new and improving existing treatment protocols for *H. pylori*‐associated GC. Administration of the gastric acid inhibitors Omeprazole and Ranitidine following *H. pylori* infection was shown to improve the healing of macroscopic inflammatory lesions, indicating that partial acid blockade is effective at reducing disease pathology in mice.^79^ The effect of long‐term administration of the anti‐inflammatory drugs Nimesulide (Cox‐2 inhibitor) to *H. pylori*‐infected mice that were also challenged with MNU significantly lowered the incidence of gastric tumors, indicating that *H. pylori*–associated inflammation was responsible for promoting gastric carcinogenesis in this model. Interestingly, Nimesulide treatment also markedly reduced atrophic gastritis.^80^

Therapeutic targeting of ulcers with Rebamipide has had positive effects on gastritis after long‐term treatment. Notably, Rebamipide decreased expression of ICAM1, HCAM and MMPs, and other inflammation related proteins, and decreased NF‐κB‐dependent DNA‐binding activity accordingly.^80^ Together, these results support the concept that long‐term administration of these anti‐inflammatory molecules may reduce the risk of GC development in *H. pylori*‐infected individuals.

Although antibiotic treatment clearly shows effectiveness in controlling *Helicobacter* infection in mice, the response in a specific preclinical model is likely to be affected by compounding mutations and genetic polymorphisms carried by the host. *H. pylori* infection of p27‐deficient mice, for instance, revealed that a combination treatment regime of Omeprazole, Metronidazole, and Clarithromycin controlled infection equally well irrespective of whether the treatment occurred 15 or 45 weeks post‐infection, with both groups scoring similarly for inflammation, epithelial defects, metaplasia, and atrophy.^81^ By contrast, *H. pylori* eradication in INS‐GAS mice was more effective when therapeutic intervention was administered at an early point of infection.^58^

The emergence of antibiotic resistance against *H. pylori* has spurred considerable efforts to develop small molecule inhibitors with high specificity against bacterial virulence factors. For example, treatment of *H. pylori*‐infected mice with a small molecule inhibitor against CagA impaired gastric colonization and alleviated disease.^82^ Other targets include pathways required by the bacterium to survive, including the metabolic conversion of urea to ammonia and bicarbonate by‐products. Accordingly, treatment of infected mice with the urease inhibitor Fluorofamide significantly decreased *H. pylori* colonization.^83^ In another study, the small molecule inhibitor HPi1 reduced *H. pylori* colony counts to below detectable limits.^84^ Collectively, these results validate mouse models for *Helicobacter* infection as important platforms for the development and testing of potential therapeutic candidates against this disease.

## Genetic and chemically induced models of premalignant states

**Figure 3:**
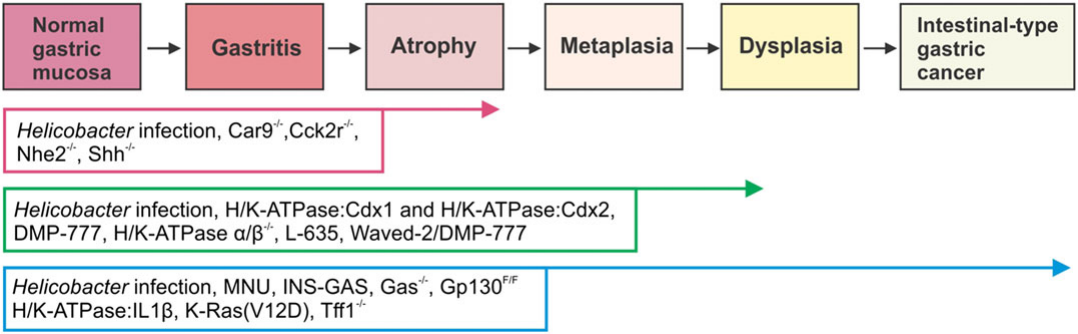
Chemical and genetic models of gastric cancer (GC). GC is preceded by a series of discrete stages that include chronic gastritis, atrophy, metaplasia, and dysplasia. In addition to chemically and genetically induced models of GC (blue boxes), mice that develop atrophy (pink boxes) and metaplasia (green boxes) have also been developed and used to determine if early intervention at these stages may assist in treatment and prevention of GC. These mouse models exhibit manipulation of genes that result in overexpression or deficiency in growth factors (including hormones and cytokines), as well as mutations in tumor suppressors and oncogenes.

Intestinal‐type GC is preceded by a sequence of histologically discrete stages that include chronic gastritis, atrophy, metaplasia, and dysplasia. In addition to mouse models of GC, several models of atrophy and metaplasia have also been developed to determine if early intervention at these stages may assist in GC prevention. Here, we focus on some models of atrophy and metaplasia including the commonly used H/K‐ATPase transgenic mutant mice (Fig. 3) and also briefly outline the histopathological characteristics of other established models in Table 3.

**Table 3:** Mouse models of gastric atrophy and metaplasia

Model	Onset	Phenotype	Ref.
CAG‐BTC	4 months	Atrophy	Dahlhoff *et al*.^99^
Car9^−/−^	1 month	Atrophy	Gut *et al*.^91^
Cck2r^−/−^	4 months	Atrophy	Nagata *et al*.^168^
H/K‐ATPase:Cdx1 and H/K‐ATPase:Cdx2	4 months	Metaplasia	Mutoh *et al*.^95^ and Silberg *et al*.^92^
DMP‐777	1–2 weeks	Atrophy + SPEM	Goldenring *et al*.^100^
†H/K‐ATPase‐*α* ^−/−^	3 months	Atrophy + IM	Spicer *et al*.^88^ and Judd *et al*.^89^
†H/K‐ATPase‐*β* ^−/−^	17 days	Atrophy + IM	Scarff *et al*.^90^ and Franic *et al*.^170^
†H/K‐ATPase‐*β*/DT	1–11 months	Atrophy	Li *et al*.^8^
H/K:Ifn‐*γ*	5–15 months	SPEM	Syu *et al*.^171^
Shh^−/−^	3–8 months	Atrophy	Xiao *et al*.^172^
L‐635	1 week	Atrophy + SPEM	Nam *et al*.^173^
Nhe2^−/−^	17 days	Atrophy	Schultheis *et al*.^174^
MT:Tgf‐*α*	3 months	Atrophy	Takagi *et al*.^97^
Waved‐2 + DMP‐777	1 week	SPEM	Ogawa *et al*.^102^

† Commonly used model

DT, diphtheria toxin; Ref., references; SPEM, spasmolytic polypeptide expressing metaplasia.

### H/K‐ATPase transgenic and knock‐out mutants

The secretion of hydrochloric acid in the stomach is dependent on H/K‐ATPase, a heterodimeric enzyme localizing to the tubulovesicular and canalicular membranes of parietal cells.^85^ H/K‐ATPases are required for parietal cell viability and normal development of the gastric mucosa, because H/K‐ATPase inhibition with Omeprazole results in parietal cell degeneration and expansion of pre‐parietal cell compartments.^86^ The latter results were also replicated following parietal cell ablation through transgenic expression of the diphtheria toxin under the control of the H/K‐ATPase gene promoter.^8^, ^87^ Given that the loss of mature parietal cells in these models also resulted in a concomitant loss of chief cells and an increase in progenitor cells, H/K‐ATPases may also have a function to maintain gastric gland homeostasis by regulating the differentiation and maturation of other epithelial cell types in the stomach.

Surprisingly, the two subunits of H/K‐ATPase have both overlapping as well as non‐overlapping functions, which have been revealed by genetic ablation studies. While *α*‐subunit‐deficient and *β*‐subunit‐deficient strains both develop achlorhydria and hypergastrinemia, loss of the *β*‐subunit selectively also results in gastric mucosal hypertrophy and a reduced number of chief cells.^88^, ^89^, ^90^ Meanwhile, loss of the *α*‐subunit confers an age and gender‐dependent (female‐biased) gastric hyperplasia with incomplete intestinal metaplasia from 3 months of age, which is associated with a significant increase in *RegIIIγ* and *RegIIIδ* mRNAs.^89^

### Car9^−/−^ mice

Carbonic anhydrase is present in the basolateral membranes of gastrointestinal epithelial cells where it regulates acid–base balance and intercellular communication.^91^ It is aberrantly expressed in many carcinomas including GC. Mice with null mutations of the corresponding *Car9* gene develop gastric hyperplasia of the glandular epithelium by 1 month of age, which is characterized by the overproduction of mucous‐secreting pit cells and the depletion of pepsinogen‐positive chief cells.^91^ While the hyperplastic phenotype of Car9^−/−^ mice shares many features of hyperplasia observed in mice lacking the *β*‐subunit of H/K‐ATPase, this model also differs in several aspects. For instance, unlike H/K‐ATPase:*β* ^−/−^ mice, Car9^−/−^ animals do not develop hypergastrinemia and hypochlorhydria. Because Car9^−/−^ mice show normal gastric acidity, this suggests that the *Car9* gene plays a more extensive role in regulating differentiation and proliferation of gastric mucosal cells, rather than being limited to acid production.

### Cdx1 and Cdx2 transgenic mice

Cdx1 and Cdx2 are intestine‐specific transcription factors that belong to the caudal‐related homeobox gene family. In the normal intestine, Cdx2 is expressed in differentiated epithelial cells of the villi (i.e. in absorptive enterocytes, goblet, and enteroendocrine cells), while Cdx1 expression is predominately localized to proliferating cells towards the base of intestinal crypts.^92^, ^93^ Although the normal gastric mucosa does not express Cdx1 and Cdx2, strong nuclear immunoreactivity is thought to molecularly underpin intestinal metaplastic transformation observed in the gastric mucosa of patients.^94^ Indeed, enforced expression of Cdx1 or Cdx2 in the glandular epithelium of the stomach using the H/K‐ATPase *β*‐subunit gene promoter leads to the development of intestinal metaplasia in transgenic mice.^92^, ^93^ Interestingly, intestinal metaplasia in H/K‐ATPase:Cdx2 transgenic mice is characterized by the appearance of absorptive enterocytes, goblet, and enteroendocrine cells, while H/K‐ATPase:Cdx1 transgenic mice exhibit intestinal metaplasia comprising all four intestinal epithelial cell types.^95^ Moreover, pseudopyloric gland metaplasia is only observed in H/K‐ATPase:Cdx2, but not in H/K‐ATPase:Cdx1 animals, and the proliferating cell nuclear antigen shows scattered expression throughout the metaplastic areas H/K‐ATPase:Cdx1 mice rather than the staining at the base of the glands observed in H/K‐ATPase:Cdx2 animals.^95^ Collectively, these results indicate that Cdx1 and Cdx2 are both able to induce intestinal metaplasia albeit with subtle differences in cellular differentiation and proliferation.

### EGFR dependent hyperplasia: TGFα and BTC transgenic mice

Ménétrier's disease is a potentially premalignant human gastric disorder characterized by hypertrophy of the gastric mucosa, atrophy of the glandular compartment, foveolar hyperplasia, and cystic dilation of gastric glands.^96^ Ménétrier's disease patients show increased expression of the epidermal growth factor receptor (EGFR) ligands transforming growth factor‐alpha (TGF*α*) and betacellulin (BTC) and therefore can be treated with the anti‐EGFR monoclonal antibody Cetuximab.^96^ Indeed, 3‐months‐old mice that were engineered to overexpress TGF*α* in the fundic mucosa develop foveolar hyperplasia, characterized by achlorhydria and excessive mucous production.^97^, ^98^ Likewise, BTC overexpression results in severe age‐dependent hyperplasia of the foveolar epithelium with the formation of large cysts and extensive depletion of the gastric mucosa.^99^

### DMP‐777 treatment

DMP‐777 is a potent leukocyte elastase inhibitor, which also acts as a parietal cell‐specific proton translocator to facilitate proton exchange across lipid bilayers. However, high doses of DMP‐777 have also been shown to damage membranes of acid‐secretory vesicles in parietal cells and promote hypochlorhydria and increased serum gastrin levels.^100^ After 3 days of DMP‐777 exposure, mice develop acute oxyntic atrophy, which precedes prominent foveolar hyperplasia. By 7–10 days, SPEM develops in the fundus, suggesting that the induction of gastric metaplasia is a direct consequence of parietal cell loss. Interestingly, withdrawal of DMP‐777 leads to complete restitution of the normal mucosa within 3 months, consistent with the capacity of parietal cells to maintain normal gastric mucosal functions.^100^ Because DMP‐777 inhibits neutrophil elastase, and therefore prevents the accumulation of inflammatory cell infiltrates, long‐time administration does not result in dysplasia even in the presence of severe oxyntic atrophy and SPEM.^100^ It is likely that DMP‐777 during inflammation promotes tumor progression following an oncogenic mutation in susceptible epithelial cells.

The use of DMP‐777 in genetically modified mouse models has been helpful to better understand the contributions of gastrin,^101^ EGFR ligands, and other signaling molecules to SPEM.^102^, ^103^ While continuous DMP‐777 treatment of wild‐type mice rapidly induced foveolar hyperplasia, hypergastrinemia and SPEM as described previously, a single dose of DMP‐777 in gastrin‐deficient mice was sufficient to induce oxyntic atrophy and SPEM in the absence of foveolar hyperplasia.^101^ Thus, the lack of gastrin is likely to promote SPEM in response to oxyntic atrophy by trans‐differentiation of chief cells.^101^ Likewise, DMP‐777 treatment of Waved‐2 mice carrying a hypomorphic mutation in the EGFR tyrosine kinase (Egfr^wa2^), or mice deficient for the EGFR ligand amphiregulin, accelerated the development of SPEM.^102^, ^103^ Interestingly, loss of TGF*α* did not influence SPEM induction, indicating that specific EGFR ligands play key roles in regulating the induction of metaplasia in DMP‐777 treated mice.^103^

## Genetic models of GC

Genetically engineered mice have helped to elucidate the contribution of mutations in the neoplastic gastric epithelium and the surrounding host tissue (Fig. 3). These models have exploited standard recombinant DNA technology to modify the germline of mice to achieve overexpression or deficiency in growth factors (including hormones and cytokines and their receptors), as well as mutations in tumor suppressors and oncogenes. Here, we discuss some of the most commonly used mouse models of GC including the INS‐GAS, H/K‐ATPase:*IL‐1β*, and Gp130^F/F^ mutant mouse strains, as well as briefly summarize the histopathology of a selection of others (Table 4).

**Table 4:** Genetic mouse models for GC

Model	Onset	Incidence, %	Location	Type	Inv.	Met.	Ref.
ACT‐GAS	20 months	100	Corpus	WD	−	−	Kanda *et al*.^175^
CA‐AhR	12 months	100	Corpus	WD	+	+	Andersson *et al*.^176^
CEA‐SV40	7 weeks	100	Antrum	WD	+	−	Thompson *et al*.^177^
Atp4b‐Cre; Cdh1^loxP/loxP^; p53 ^loxP/loxP^	12 months	100	Corpus	PD	+	+	Shimada *et al*.^178^
Cdh1^+/−^ + MNU	10 months	46	Antrum	PD	−	−	Humar *et al*.^34^
H/K‐ATPase:Cdx2	3 months	100	Corpus	WD	+	−	Mutoh *et al*.^179^
Gas^−/−^	12 months	60	Antrum	WD	−	−	Zavros *et al*.^9^
GB‐Cre;Smad4^Floxed^	18 months	100	Antrum	WD	−	−	Hahn *et al*.^180^
Gp130^F/F^	3 months	100	Corpus + antrum	WD	−	−	Ernst *et al*.^126^
INS‐GAS	20 months	75	Corpus	WD	+	−	Fox *et al*.^108^
K19:Kras‐(V12D)	16 months	38	Corpus	WD	+	−	Okumura *et al*.^143^
H/K‐ATPase:IL1*β*	18 months	30	Antrum	WD	−	−	Tu *et al*.^119^
Runx3^−/−^ + MNU	1 year	60	Corpus + antrum	WD	−	−	Ito *et al*.^33^
Lgr5^CreERT2;^APC^Floxed^	3 weeks	100	Antrum	WD	−	−	Barker *et al*.^181^
MMTV‐Ad12	4 months	56	SCJ	WD	+	+	Koike *et al*.^182^
Mth1^−/−^	18 months	13	Antrum	WD	−	−	Tsuzuki *et al*.^183^
Smad3^−/−^	10 months	100	SCJ	WD	+	−	Nam *et al*.^184^
Smad4^+/−^	18 months	100	Corpus + antrum	WD	+	−	Takaku *et al*.^185^
Tff1^−/−^	5 months	30	antrum	WD	+	−	Lefebvre *et al*.^151^
K19*‐*Wnt1:C2mE	5 months	100	SCJ	WD	+	−	Oshima *et al*.^186^
Villin‐Cre;KLF4^Floxed^	20 months	29	Antrum	WD	−	−	Li *et al*.^35^

+, present; −, absent; GC, gastric cancer; Inv., invasion; Met., metastasis; PD, poorly differentiated; Ref., references; SCJ, subcolumnar junction; WD, well differentiated.

### Gastrin mutants

Gastrin is a hormone produced by G cells in the antral mucosa, which regulates acid secretion in response to food intake, maintains gastric architecture, and promotes homeostatic renewal of the gastric epithelium.^104^ Accordingly, gastrin regulates cell division, invasion, angiogenesis, and apoptosis.^105^ Therefore, mouse models were developed to either excessively express gastrin (in INS‐GAS transgenic mice) or to completely ablate gastrin expression (in Gas^−/−^ knockout mice).

#### INS‐GAS mice

INS‐GAS mice express human gastrin under the control of the insulin promoter yielding a twofold increase of amidated gastrin in the serum of these mice.^106^ In the absence of *Helicobacter* infection, INS‐GAS mice show an increase in gastric acid secretion and parietal cell mass at 1–3 months of age and eventually develop atrophy as a result of hypergastrinemia. However, *H. pylori*‐infected INS‐GAS mice develop intramucosal carcinomas with submucosal and intravascular invasion by less than 1 year, which subsequently progress to tumors in the gastric corpus of 20‐month‐old mice.^107^, ^108^ Interestingly, gender is a major modifier of *H. pylori*‐associated GC in INS‐GAS mice, with males exhibiting a higher incidence than females.^75^ This gender bias is attributed to estrogen, because ovariectomized female INS‐GAS mice are equally susceptible to *H. pylori*‐induced gastric neoplasia as their male littermates.^109^ Furthermore, GC development in INS‐GAS mice is also influenced by genetic background, because all of the aforementioned observations are only made in mice on the FVB genetic background, while on the C57BL/6 background even *Helicobacter*‐infected INS‐GAS mice only develop hyperplasia and dysplasia in the corpus region.^110^

Due to the extensive latency period of *H. pylori* infection‐dependent induction of gastric atrophy and GC in wild‐type mice, such studies are frequently performed with INS‐GAS mice to accelerate GC, and to study the effect of compounding gene mutations in the host,^111^ of the microbiota,^108^ and gender.^109^ Conversely, this model has also served to identify potential prophylactic strategies for GC. Combination treatment with the nonsteroidal anti‐inflammatory drug Sulindac and the triple antibiotic (Omeprazole, Metronidazole, and Clarithromycin) *H. pylori* eradication therapy was beneficial for reducing the production of pro‐inflammatory cytokines in the stomach and preventing the progression from severe dysplasia to GC.^112^ Similarly, *H. felis*‐infected INS‐GAS mice treated with the gastrin receptor antagonist YF476, or with the histamine receptor antagonist Loxitidine, resulted in partial suppression of gastric acid secretion and neoplastic progression, which was further reduced when combining the two drugs.^113^ These latter findings support an important role for the gastrin–histamine axis in *Helicobacter*‐induced gastric carcinogenesis and raise the possibility for this drug combination (YF476 and Loxitidine) to serve as an alternative clinical approach for long‐lasting acid suppression. Given the male bias for *H. pylori*‐associated GC incidence, Ins‐Gas mice have also been used to establish that estrogen supplementation protects male mice against gastritis and premalignant gastric lesions.^109^

#### Gas^−/−^ mice

Hypogastrinemia is an established risk factor for the development of gastric ulcers, and the associated achlorhydria is likely to predispose GC. On a C57BL/6 background, gastrin knockout (Gas^−/−^) mice exhibit mild changes in gastric architecture, characterized by a decrease in parietal and enterochromaffin‐like cells.^105^ However, when crossed onto the 129/Sv background, achlorhydria results in bacterial overgrowth and chronic inflammation.^114^ On an FVB background, 1‐year‐old Gas^−/−^ mice develop spontaneous antral tumors, which can be accelerated following exposure to MNU.^9^, ^37^ Interestingly, antibiotic treatment of young GAS^−/−^ mice on an 129/Sv background reversed fundic and antral hyperplasia,^114^ while exogenous gastrin administration temporarily prevented both intestinal metaplasia and the upregulation of an interferon‐dependent transcriptional response.^115^ However, progressive and irreversible intestinal metaplastic changes eventually develop in aging C57BL/6 Gas^−/−^ mice and result in tumor development.^115^ Given the differences in susceptibility and disease onset between genetic backgrounds, these results reaffirm the importance of choosing the appropriate strain when considering the use of these models for studying GC pathogenesis.

Although the molecular mechanisms by which gastrin deficiency promotes GC remain unclear, several mechanisms have been proposed. Because achlorhydria enables enterococal overgrowth in Gas^−/−^ mice, these bacteria can produce carcinogenic by‐products such as N‐nitrosamines through the metabolism of nitrates.^114^, ^115^ In support of this, increased production of N‐nitrosamines is also observed in atrophic gastritis patients with achlorhydria, which is consistent with their increased risk for GC.^116^ Secondly, because bacterial infection initiates an inflammatory response associated with increased expression of IFN‐*γ* and other cytokines, bacterial overgrowth is likely to alter gastric gene expression and promote tumor formation.^115^ One such example is the inverse correlation between Cdx2 activation and luminal acidity in the duodenum.^117^ The increased activation of transcription factors such as Cdx2 and tumor development correlates with achlorhydria severity in the stomach.^115^ Thus, gastrin deficiency in Gas^−/−^ mice is most likely to facilitate malignant transformation by exacerbating achlorhydria in the stomach. Comparison between the INS‐GAS and Gas^−/−^ models has also revealed distinct roles for gastrin in distinct anatomical sections of the stomach, because tumor development is restricted to the corpus and antrum of these mice, respectively, and MNU‐treated INS‐GAS mice do not readily develop antral tumors.^37^ Collectively, these findings highlight the importance of considering the corpus and antrum as distinct anatomical sites when characterizing tumor pathology in mouse models.

### H/K‐ATPase:IL‐1***β*** transgenic mice

IL‐1*β* is a pleiotropic inflammatory cytokine regulating cellular proliferation, differentiation, and apoptosis. IL‐1*β* is excessively expressed in response to *H. pylori* infection,^118^ and polymorphisms in *IL‐1β* gene have been linked to increased risk of *H. pylori*‐associated gastric carcinogenesis.^47^, ^48^ Experimental insights into the role of IL‐1*β* during epithelial homeostasis have largely been gained from mice that express constitutive IL‐1*β* activation. These H/K‐ATPase:IL‐1*β* transgenic mice spontaneously develop inflammation, atrophy, metaplasia, and gastric adenocarcinomas in 30% of mice by 18 months of age.^119^ When infected with *H. felis*, tumor development in these mice is accelerated and occurs within 1 year of age, thereby confirming a link between elevated IL‐1*β* and an increased risk for inflammation‐associated GC.^119^

Several mechanisms underpinning IL‐1*β*‐associated GC have been identified using the IL‐1*β* transgenic mouse model. These include excessive activation of the NF‐κB pathway, resulting in elevated levels of pro‐inflammatory cytokines such as IL‐1*β*, IL‐6, IFN‐*γ*, and TNF*α*,^119^ as well as an increased recruitment of myeloid‐derived suppressor cells to the gastric epithelium where they suppress the host's anti‐tumor immune response.^120^ IL‐1*β* has also been shown to promote gastric atrophy by suppressing Sonic hedgehog gene expression in parietal cells, resulting in decreased acid secretion and release of intracellular calcium.^121^

The IL‐1*β* transgenic mouse has substantially contributed to our understanding of the role of stromal cells including cancer‐associated fibroblasts (CAFs) in gastric tumorigenesis. Although it was widely accepted that the interaction between tumor cells and CAFs promotes tumorigenesis, the precise role of these cells remain unclear. However, in this model, CAFs accumulated during chronic inflammation, particularly during dysplasia, and these CAFs were also *α*SMA‐positive, a marker of myofibroblasts, and derived from the bone marrow.^122^ During chronic inflammation and tumorigenesis, these *α*SMA‐positive CAFs expanded and migrated in a TGF‐*β*‐dependent and SDF‐1α‐dependent manner to incipient tumor cells where they contributed to and sustained tumor growth by expressing IL‐6, Wnt5*α*, and BMP4.^122^ In another study, the role of T‐cells in GC was analyzed to show that IFN‐*γ* overexpression inhibited gastric carcinogenesis because of suppression of Th1 and Th17 immune responses by IFN‐*γ* through Fas induction and apoptosis of CD4 T‐cells.^123^

### Gp130^F/F^ mice

Increased activation of the latent transcription factor STAT3 is frequently observed in many solid human malignancies including GC.^124^ However, while excessive STAT3 signaling promotes upregulation of genes involved in angiogenesis, cell‐cycle progression, and cell survival, the complex role of STAT3 during tumor progression is still incompletely understood.^124^, ^125^ One approach to explore the role of excessive STAT3 activity in response to the IL‐6 family of cytokines exploited a phenylalanine knock‐in substitution at tyrosine 757 in the cytoplasmic domain of the IL‐6 receptor *β*‐chain Gp130, which prevents binding of the STAT3 negative regulator suppressor of cytokine signaling (SOCS)3.^126^ Accordingly, the homozygous presence of this mutation in the corresponding Gp130^F/F^ mutant mice facilitates excessive activation of STAT3 and to a lesser extent of STAT1.^127^ Gp130^F/F^ mice develop splenomegaly and rapid tumor development in the epithelium of the glandular stomach independent of *H. pylori* infection and highlight a key role for STAT3 signaling in promoting gastric tumorigenesis.^126^ Alongside STAT3 activation, excessive activation of the mTOR complex 1 (mTORC1) has also been reported in Gp130^F/F^ mice, while administration of an mTORC‐1 inhibitor suppressed tumor development and progression.^128^ Interestingly, gp130‐dependent activation of mTORC1 was found to occur via PI3K/AKT and require Janus kinases (JAK), but neither STAT3 nor phosphorylation of tyrosines on gp130.^128^ Because tumors in Gp130^F/F^ mutants resemble well‐differentiated human gastric adenocarcinomas, these results indicate an essential role for the PI3K/mTORC1 pathway in inflammation‐associated tumorigenesis that could be therapeutically exploited.

The development of tumors in Gp130^F/F^ mutants follows the classical intestinal‐type developmental sequence, although these lesions rarely become invasive and never metastasize to distant organs. Corpus hyperplasia in Gp130^F/F^ mice occurs from 14 weeks of age, while antral tumors can be observed from 6 weeks onwards.^126^ In addition to increased expression of anti‐apoptotic and pro‐angiogenic genes,^129^, ^130^ excessive STAT3 activation also correlates with increased *Tlr2* expression in these tumors, while genetic ablation of *Tlr2* alleviates gastric tumor formation independent of inflammation.^131^ Interestingly, *TLR2* expression is elevated in *H. pylori*‐positive patients, and *TLR2* polymorphisms are associated with an increased risk for GC.^132^, ^133^, ^134^ TLR2 signaling is thought to involve the adapter proteins MyD88 and Mal, which promote activation of NF‐*κ*B, p38, and MAPK pathways. Interestingly, genetic ablation of *Myd88* in Gp130^F/F^ animals suppressed tumorigenesis to a level comparable with Gp130^F/F^;Tlr2^−/−^ mice, while the tumor burden of Gp130^F/F^;Mal^−/−^ compound mutants remained similar to that of their Gp130^F/F^ littermates.^135^ Given the interest in developing small molecular inhibitors against TLR2 for human GC,^136^, ^137^ the use of the Gp130^F/F^ mouse as a model for the testing of anti‐TLR2 molecules has already shown favorable results.^131^

In addition to providing insight into the mechanisms of STAT3‐mediated tumorigenesis, the Gp130^F/F^ mouse model has also served as an important tool for the identification and validation of novel therapeutics against STAT3 signaling. One example is the targeting of gp130‐associated JAK kinases, which phosphorylate cytoplasmic STAT3 to promote its dimerization, nuclear translocation, and transcriptional activity.^138^ Accordingly, systemic administration of the small molecule JAK1/2 inhibitor AZD1480 attenuated tumor progression in Gp130^F/F^ mice, which was associated with reduced STAT3 activation, diminished tumor cell proliferation, and increased apoptosis.^139^ Likewise, treatment of Gp130^F/F^ mice with the JAK inhibitor WP10336 for 2 weeks reduced gastric tumor volume by 50% and correlated with reduced JAK2 and STAT3.^140^ Although these results provide a strong rationale for pharmacological targeting of JAK kinases, systemic inhibition of JAK2 in mice and humans can also cause thrombocytopenia and other undesired on‐target side‐effects.^141^ Given the prominent role of IL‐11 in the development and progression of inflammation‐associated GC, this cytokine has raised considerable interest as a potential therapeutic target associated with fewer adverse effects.^127^ In particular, we have shown that treatment with an IL‐11 antagonist, IL‐11 Mutein, reduced STAT3 activation and significantly inhibited tumor development and gastric epithelial hyperplasia in Gp130^F/F^ mice.^127^ Importantly, IL‐11 Mutein treatment for 4 weeks did not result in side‐effects, in particular, when assessing hematopoietic parameters,^127^ supporting IL‐11 signaling as a potential therapeutic target for GC.

### K‐Ras transgenic mice

The small GTP‐binding protein K‐Ras belongs to the superfamily of RAS‐like GTPases and regulates cell‐cycle progression, apoptosis, and senescence and activates somatic mutations in *K‐RAS* lock the protein in a GTP‐bound, permanently active form.^142^ While less prevalent than other mutations, activating mutations in *K‐RAS* have also been observed in GC, and these have been shown to promote proliferation and tumor cell invasiveness.^142^

The role of K‐Ras in the pathogenesis of GC has been explored with several oncogenic K‐Ras mutant mouse strains. One model exploits the K19‐promoter to constitutively express the oncogenic K‐Ras(V12D) gene. These K‐Ras(V12D) transgenic mice exhibit metaplasia, dysplasia, and adenocarcinoma formation in 38% of the mice by 16 months of age, correlating with enhanced recruitment of inflammatory cells and fibroblasts, as well as elevated levels of CXCL1 and other chemokines.^143^ In contrast, when the K‐Ras(G12D) mutation is targeted to the gastric epithelium, hyperplasia, metaplasia, and adenocarcinoma are observed.^144^ Interestingly, ubiquitous K‐Ras activation in K‐Ras(G12D/+) mice results in accelerated inflammation, hyperplasia, and metaplasia in the stomach, although neoplastic changes are not observed.^145^ Together, these results suggest that oncogenic mutations in K‐Ras are required in a specific cell type for the progression from tumor to cancer.

### Tff1^−/−^ mice

Trefoil factor 1 (TFF1) is a protein that appears to confer a tumor suppressor role and is predominantly expressed by surface mucosal cells of the stomach where it regulates the normal differentiation of gastric glands.^146^ Through its interaction with various mucins, TFF1 promotes correct organization of the mucous layer and protection of the gastric mucosa from erosion.^147^ In support of this, TFF1 expression increases in inflamed and damaged gastrointestinal tissues,^148^, ^149^ and iFABP:pS2 mice overexpressing the Tff1/pS2 transgene exhibit an increased resistance to ulceration.^150^ By contrast, Tff1^−/−^ mice develop glandular hyperplasia characterized by crypt elongation and nuclei enlargement associated with loss of cell polarity from 3 weeks of age, and invasive adenocarcinomas are observed in 30% of mice at 5 months.^151^ In support of the role of inflammation in gastric carcinogenesis, loss of *Tff1* promotes activation of the canonical NF‐*κ*B signaling pathway and results in increased immune cell infiltration into the submucosa and glandular epithelia.^152^ Tumors from Tff1^−/−^ mice also express elevated levels of COX‐2,^153^ which has been suggested to indicate reduced survival of human GC patients.^154^ However, whereas COX‐2 is similarly expressed in hyperplastic gastric tissue and gastric adenomas of Tff1^−/−^ mice, COX‐2 expression increases with disease severity in human precursor lesions.^155^ In Tff1^−/−^ animals COX‐2 expression appears to be restricted to stromal cells, whereas in human GC patients, COX‐2 is mostly associated with epithelial cells.^155^, ^156^ Nevertheless, early treatment with the COX‐2 inhibitor Celecoxib has been shown to reduce adenomatous growth in Tff1^−/−^ mice, while treatment of mice with fully developed adenomas led to a significant reduction in tumor multiplicity and size.^157^ However, withdrawal of Celecoxib triggered the reappearance of adenomas,^157^ suggesting that reciprocal increase in COX‐2 activity following Tff1 deletion functions as a tumor promoting mechanism.

## Future perspectives

Although various mouse models with distinct susceptibilities to GC development and pathologies are now readily available for the study of gastric tumorigenesis, the molecular mechanisms that underpin GC development remain incompletely understood. Genome‐wide sequencing studies have been useful in identifying additional epigenetic and genetic changes that significantly impact GC development and progression. Recent sequencing studies on human gastric tumors have associated new mutations in genes such as *PSCA* 158 and *PLCE1* 159 with increased susceptibility to develop GC, while other studies have identified genes in GC subtypes associated with distinct clinical outcomes.^6^ Targeting these genes in the context of mouse models is likely to provide insights into their causal mechanism by which they affect the development and progression of GC.

One major limitation is the scarcity of models in which mutations are only targeted to specific gastric cell lineages. The use of DNA recombinases, such as Cre, will enable induction of mutations in the various gastric epithelia and evaluate the potential of these cells to give rise to solid cancers. This, however, will require the identification of promoter sequences that restrict transgene expression to the specific gastric epithelial cells. For example, drivers such as K19:Cre and Foxa3:Cre are also expressed in the colon and intestine, while a Tff1:CreERT2 driver confers recombination to the glandular epithelium of the stomach.^160^ A number of studies have identified potential stem‐cell markers for gastrointestinal tissues. However, many of these markers such as Lgr5 and Lrig^161^ are not exclusively expressed in the stomach. Other potential candidates include TFF2 and Mist1.^162^, ^163^ TFF2‐expressing cells are observed in the base of antral glands as well as the isthmus, where they give rise to mucous neck, parietal, and zymogenic cells.^163^ In contrast, *Mist1* expression is restricted to mature chief cells that have been shown to give rise to SPEM after exposure to DMP‐777 treatment or *H. felis* infection,^162^ while TFF1 has already been used as a promoter to disrupt TGF‐*β*2 signaling in the gastric mucosa^164^ and TFF2:CreERT and Mist1:Cre mice are available. Further investigation of basic gastric biology through lineage tracing studies needs to be performed to identify further epithelial cell, and potentially stem cell, markers with exclusive specificity in the stomach. These studies when combined with existing GC models would fast track the identification of potential therapies for clinical trials with life‐sustaining effects.

## Conclusion

Mouse models of GC have progressed over recent decades from chemically induced random mutagenesis, to bacterial‐induced dysplasia and more recently to recombinant DNA technology‐mediated genetic mouse models. These models have revolutionized our understanding of how genes, diet, bacteria, and host immunity affect GC formation. Furthermore, mouse models of precancerous change have shed light on the molecular mechanisms by which these changes affect early stages of carcinogenesis, aided clinical detection, and have pinpointed therapeutic strategies for intervention at the pre‐neoplastic stages. Given the large number of models available, an in‐depth understanding of their tumor histopathology and molecular signatures is important to ensure that new pre‐clinical drug studies exploit the model that is most relevant to the hypothesis investigated. Clearly, factors such as genetic background, gender, diet, and the gut microbiota associated with housing conditions impact on the various models and thus need to be carefully controlled for when interpreting results. The incorporation of current research methods such as genome/exome‐wide sequencing and lineage tracing has yielded fantastic results in the field of colorectal cancer and will provide the foundation for similar advancements on disease mechanisms and identification of therapeutic targets for clinical trials.
